# Synchronous double primary lung cancers via p53 pathway induced by heavy smoking

**DOI:** 10.4103/0256-4947.62837

**Published:** 2010

**Authors:** Cheng-Chih Lin, Chih-Feng Chian, Wann-Cherng Perng, Ming-Fang Cheng

**Affiliations:** a From the Division of Pulmonary and Critical Care, Department of Internal Medicine, Tri-Service General Hospital, National Defense Medical Center, Taipei, Taiwan; b From the Division of Pulmonary Medicine, Department of Internal Medicine, Armed-Forces Zuoying General Hospital, Kaohsiung, Taiwan; c From the Department of Pathology, Tri-Service General Hospital, National Defense Medical Center, Taipei, Taiwan

## Abstract

Differences in the histological manifestation of synchronous lung cancers are rare. Synchronous multiple primary lung cancers (SMPLC) are associated with long-term tobacco use, which could independently lead to mutations in the p53 and K-ras genes. We present the case of an 82-year-old man who smoked 30 cigarettes daily for the past 60 years. CT of the chest showed a right upper lobe mass. Bronchoscopy revealed an intra-lumen nodular lesion in the right lower lobe bronchus. The diagnoses of small cell lung carcinoma (SCLC) of the right upper lobe and non-small cell lung carcinoma (NSCLC) of the right lower lobe were confirmed by the morphologic features and the detected immunoreactivity. Immunohistochemical analyses showed a strong positive reaction for p53 in samples of the SCLC and NSCLC. The cancers had a different phenotype, but similar genetic abnormalities may have developed due to the carcinogens in the cigarettes.

It is difficult to distinguish multiple synchronous lung tumors from multicentric lung cancers, or from a single lung cancer with intrapulmonary metastases or pulmonary metastases that have originated from primary cancers in different organs. These cancers may be distinguished as a second primary lung cancer on the basis of different molecular genetic characteristics or in the absence of radiologic features of mediastinal node involvement.^1^ A synchronous second focus of lung cancer in a different lobe is easily defined as a second primary lung cancer when the two sites of tissues are different histologic types. Clinical trials have shown that the incidence of synchronous multiple primary lung cancers (SMPLC) ranges from 1% to 7%.^2^,^3^ The occurrence of synchronous lung cancers, which is defined by a second tumor in a different lobe and with a different histological manifestation, is rare.^3^–^5^ In 1953, Slaughter et al proposed that those preneoplastic and neoplastic lesions are usually multifocal and develop throughout the entire respiratory tract because of exposure to similar carcinogens. They referred to this phenomenon as the concept of “field cancerization”.^6^ Sozzi et al supported this hypothesis and showed that cytogenetic abnormalities could be detected in epithelial cells of these tumors and that these cytogenetic changes occur in cells distant from normal appearing epithelial cells.^7^

## CASE

An 82-year-old male was admitted to our hospital because of cough with blood-streaked phlegm. These symptoms had been sustained for two weeks. He had smoked 30 cigarettes per day for the past 60 years. The patient did not experience dyspnea, fever, chest pain, body weight loss, or poor appetite. His medical history and family history were unremarkable. Physical examination on admission revealed a clear breathing sound; moreover, abnormalities in neck size as well as supraclavicular lymph nodes were not detected. The results of the laboratory tests were within the normal range except for a mild normocytic anemia.

**Figure 1:**
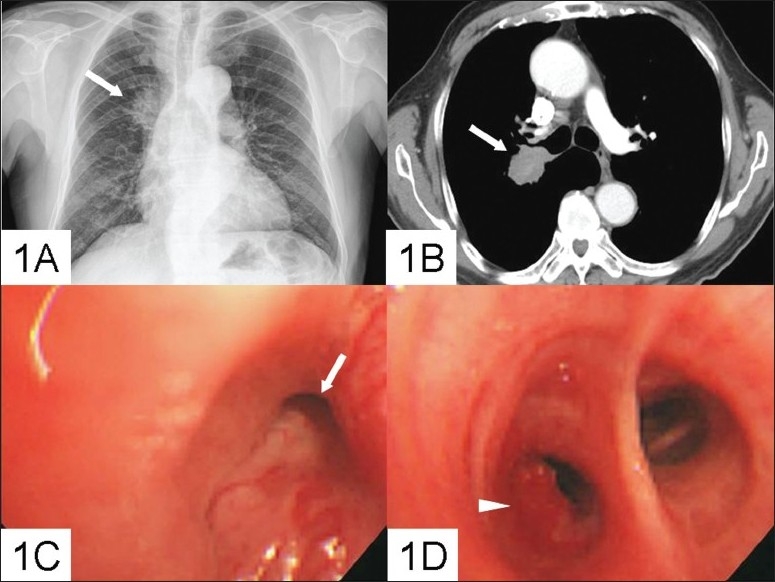
Chest radiograph showing a mass lesion on the right upper lobe (a). The contrast-enhanced CT of the chest shows a right upper lobe soft-tissue mass with encasement of the bronchus, mediastinum invasion and an enlarged right hilar lymph node (b). The bronchoscopic image shows extensive submucosal and lymphangitic infiltration with partial obstruction of the right upper lobe orifice (c) and one whitish, keratinized tumor, easy to bleed on touch and partially occluding the RB7 bronchus lumen (d).

On performing routine chest radiography, a mass lesion on the right upper lobe was discovered (Figure 1a). Contrast-enhanced CT revealed a lobulated soft-tissue mass that measured 3.7 cm in the largest dimension on the axial plane in the medial region of the right upper lobe (RUL). In addition, the image showed encasement of the RUL bronchus, mediastinal invasion, and enlarged lymph nodes in the pretracheal retrocaval space, subcarinal space, and the right hilar region (Figure 1b). Bronchoscopic examination revealed extensive submucosal and lymphangitic infiltration with partial obstruction of the right upper lobe orifice and a whitish keratinized tumor, which bled easily upon touch, and partially occluded the right anterior segment of the lower lobe bronchial lumen (RB7, right anterior segment of the lower lobe bronchial lumen) (Figure 1c, 1d).

**Figure 2:**
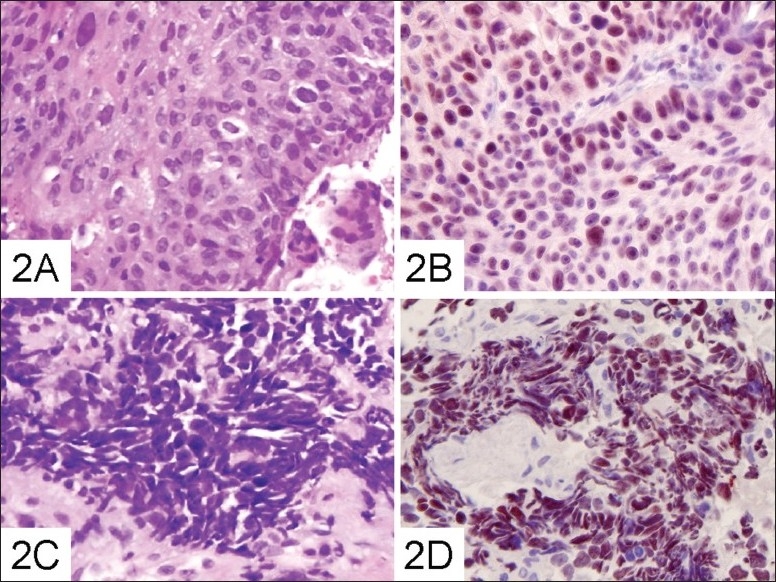
Sections of the right upper lobe lung lesion showing small cell carcinoma characterized by small, pleomorphic tumor cells with nuclear molding and “crush” artifacts (a,×400). The tumor cells were immunohistochemically positive for p53 (b,×400). The images of sections of the RB7 lung lesion show non-small cell lung carcinoma with pale to pink cytoplasm without specific differentiation (c,×400). The tumor cells were immunohistochemically positive for p53 (d,×400).

Pathologic examination of the pulmonary specimen of the RUL bronchus revealed small cell lung cancer (SCLC), which was positive for chromogranin-A and negative for CD45. The pulmonary specimen of the bronchus of RB7 showed moderately to poorly differentiated non-small cell lung carcinoma (NSCLC). In addition, the morphological features and immunohistochemical results of the tumor cells from the two distinct regions of the right lung were different (Figure 2). These results were not consistent with the characteristics reported for metastatic cancer of pulmonary origin. However, both pathologic specimens showed a strong positive reaction for p53, implying that similar carcinogenesis caused these two different types of lung cancers. We also performed an abdominal sonography and a Tc-99m whole body bone scan. The findings were unremarkable. The patient refused further examinations, was discharged and was not available for a follow-up visit.

## DISCUSSION

The occurrence of SMPLC has been reported to be approximately 1%.^8^ Small differences with regard to the etiology and clinical manifestation of SMPLC are difficult to detect due to the few number of cases; hence, there have been no reports on a definite etiology and clinical manifestations of SMPLC. In 1999, Wang et al demonstrated that patients with SMPLC had significantly more tobacco exposure. In addition, they reported that these tumors developed independently due to mutations in the p53 and K-ras genes, which suggested that field cancerization may be an important aspect of lung carcinogenesis.^9^

The etiology of lung cancer is strongly associated with cigarette smoking. Exposure to carcinogens found in tobacco causes various genetic alterations in the genome of susceptible pulmonary cells, indicating molecular tumorigenesis during multistep tumor progression.^10^ The most frequent genetic abnormalities occur in tumor suppressor genes (TSGs). The TSG p53 regulates both cell cycle progression and apoptosis. It plays a key role in protecting cells from duplicating damaged DNA. Increased expression of mutant p53 induces failure of apoptosis. This is accompanied by uncontrolled cell proliferation leading to neoplastic transformation. The TSG p53 is mutated in more than 90% of SCLCs and more than 50% of NSCLCs. In our case, specimens of SCLC and NSCLC were immunohistochemically positive for p53, suggesting that although their phenotypes are different, a similar genetic abnormality caused by carcinogens of cigarette smoking may have led to the development of SCLC and NSCLC. A recent study analyzed 70 lung tumors from 30 patients with concordant genetic alteration examination, and concluded that the majority of multifocal lung cancers had a common clonal origin. This finding may support the presentation of SMPLC in our case.^11^

Multiple synchronous tumors of histologically different cell types should be considered as separate primary lung cancers and should be staged separately.^12^ Mediastinoscopy and systemic staging are recommended in order to exclude the possibility that one pulmonary lesion metastasized from the other and that both lesions represent systemic metastases originating from a different tissue. The 5-year survival rate of patients diagnosed with SMPLC has been reported to be significantly lower than in patients with single primary tumors.^13^ The poorer prognosis for multiple cancers may be explained by the different biological features. In addition, some studies suggest that surgery should not be performed if the tumors have advanced beyond stage II.^3^,^14^ However, Chang et al proposed that multiple primary lung cancers could be regarded as a local disease rather than a systemic disease, and that they are potentially curable, especially with no metastatic lymph nodes.^15^ In 2007, Trousse also suggested that patients with SMPLC are expected to benefit from surgery after an appropriate selection process (node-negative patients with adequate pulmonary reserve).^16^ In patients with multiple synchronous lesions, limited surgical procedures to remove the synchronous lesions are preferred, especially since pneumonectomy has been associated with an increased risk of death.^16^

In conclusion, SMPLC is a rare clinical entity and the mechanism of carcinogenesis is still unknown. Heavy smoking resulting in carcinogenesis via the induction of the p53 mutation may play a role in SMPLC. Therefore, studies with large sample sizes are warranted.
